# Editorial: The Application of Nanoengineering in Advanced Drug Delivery and Translational Research

**DOI:** 10.3389/fbioe.2022.886109

**Published:** 2022-03-24

**Authors:** Zhidong Chen, Xu Chen, Gan Liu, Kai Han, Jingxiao Chen, Junqing Wang

**Affiliations:** ^1^ School of Pharmaceutical Sciences (Shenzhen), Sun Yat-sen University, Shenzhen, China; ^2^ Department of Pharmaceutical Sciences, University of Michigan, Ann Arbor, MI, United States; ^3^ Key Laboratory of Carbohydrate Chemistry and Biotechnology, Ministry of Education, School of Life Sciences and Health Engineering, Jiangnan University, Wuxi, China

**Keywords:** nanoengineering, drug delivery, cancer, immunotherapy, exosomes, osteogenesis

In recent years, nanoengineering has been playing a significant role in drug delivery and translational research as it has the potential of resolving the translational challenges of traditional therapeutic agents, such as poor solubility, side effects, limited blood-circulation time, etc. Lots of research are focusing on developing novel drug delivery systems for translation with nanoengineering, including many types like micelles (Ghosh and Biswas, 2021), liposomes (Crommelin et al., 2020), lipid nanoparticles (Wang et al., 2020), nanodiscs (Kuai et al., 2017; Xu et al., 2022), hydrogel (Daly et al., 2020), and exosomes (Kalluri and LeBleu, 2020). Some results from clinical trials have shown the efficiency of nanoengineering (Martin et al., 2020). However, many problems remain to be solved, such as immune-related adverse events (Martins et al., 2019), nanotoxicity, and poor reproducibility. Therefore, designing advanced drug delivery systems for translation needs some novel and practical approaches with chemistry, biology, and materials science, which is also the theme of this Research Topic.

This Research Topic is provided through ten articles, including eight original research articles and two review articles. The original research articles involved multiple delivery systems with nanoengineering: exosomes, nanoparticles, black phosphorus nanosheets, and hydrogels. These nanosystems were rationally designed, showing improvement and intriguing effects compared with traditional therapeutic agents in different diseases.

There are two review articles on this Research Topic. In a review article, Zhang et al. mainly discussed the crucial role and related biological mechanisms of oxidative stress in diabetic wound healing. They summarized the progress in antioxidant therapy and related bioengineering technology. The authors initially highlighted the importance of oxidative stress in the pathology of diabetic ulcers from the four overlapping stages of wound healing, showing the oxidative stress induced by hyperglycemia will largely hinder the recovery of wound healing by widely impairing the majority of cells. Then, in consideration of the significance of oxidative stress, they summarized the recent progress of antioxidant therapy for diabetic wound healing. They pointed out the key pathways in oxidative stress and corresponding therapy and introduced several therapeutic agents, including endogenous molecules like vitamins, antioxidant enzymes, hormones, and medicinal plants. The authors also summarized the biological materials used in diabetic wound healing. Most of them can load the antioxidant agents to promote their efficiency. In another review article, Cui et al. emphasized the state-of-the-art hydrogels applied in cancer immunotherapy. As one of the most biocompatible and versatile biomaterials, hydrogels can be endowed with unique and useful properties by novel design, making a difference in cancer immunotherapy. They first introduced the characteristics and design principles of hydrogels as delivery systems from five respects, including load ability, implantability, injectability, degradability, and stimulus-responsibility. Then, the applications of hydrogels in cancer immunotherapy classified by the carrying cargos were discussed. In the end, the authors discussed the challenges, clinical application potential of hydrogels, and some Research Topic that need to be considered from the clinical aspect.

In an original study, Zhao et al. developed an exosomes-based nanoplatform prepared by adding dexamethasone (DEX) in exosomes(exos) secreted from mesenchymal stem cells (MSCs), which can be thought of as a promising drug delivery system of autoimmune hepatitis (AIH). Lots of research have shown that exosomes will typically accumulate in the liver, and those secreted from MSCs have the ability of liver regeneration. Taking the positive effects of exosomes on the liver and the delivery efficiency of exosomes into account, this research attempted to combine the therapeutic features of exosomes with the potent anti-inflammation drug DEX for a synergistic treatment toward AIH. The presence of exosomes will also relieve the side effect of DEX resulting from its accumulation in the liver. *In vivo* and *ex vivo* experiments in this article proved the accumulation of this system in the liver parenchyma, and with the *in vivo* treatment of the Con A-induced AIH mouse model, the efficiency of this system compared to free drugs was certified.

Escharotomy and infection sometimes could be severe clinical challenges. Wang et al. designed a sequential therapeutic hydrogel. Through the cross-linked by modified polyvinyl alcohol (PVA-SH/ε-PL) and benzaldehyde-terminated F127 triblock copolymers (PF127-CHO), this hydrogel is endowed with excellent properties which are beneficial for deep burn wounds, such as excellent mechanical and satisfactory wound cleaning properties. The authors also loaded bromelain and EGF into the same hydrogel in stages for wound cleaning and healing. All of the intriguing properties of these novel hydrogels were confirmed by *in vitro* and *in vivo* assays, making this kind of hydrogels an excellent candidate for deep burn injuries, especially for irregular-shaped deep burn wounds in poor medical conditions.

Chen et al. developed a novel peptide design strategy with artificial neural networks. An unnatural amino acids library was introduced to generate peptides targeting the GPR40 receptor. This strategy provides a new approach for peptide drug design and an opportunity to improve clinical translation efficiency. The screening strategy designed by the authors was based on AI and unnatural amino acids to overcome the limited diversity in traditional peptide design. Site-directed mutagenesis approach and molecular dynamics simulation were applied to optimize the interaction between the candidate peptides and GPR40 and verify the stability of the peptide-protein complex. This strategy can efficiently discover peptide drugs and improve clinical translation efficiency with a small computing resource.

Nanoengineering has been one of the most effective tools in cancer medicine, nanoengineering-based approaches can modulate the systemic biodistribution and targeted accumulation of the therapeutic agents (Nam et al., 2019). In this research topic, Bao et al. designed a novel nanoparticle coated with polypyrrole for photothermal/magnetothermal therapy against cancer. The main purpose of this study was to solve the deficiency of photothermal or magnetothermal-based hyperthermia cancer therapy by combining them with nanotechnology. The poor penetration ability of the photothermal and the deficient heating efficiency of the magnetothermal was improved by the combination of polypyrrole and Fe_3_O_4_, which also enables this system with excellent hyperthermia effects and antitumor effects under the alternating magnetic field and near-infrared at the same time. After proving the photothermal and magnetic thermal properties of this system, the cytotoxicity of this combination therapy was certified to be more efficient through *in vitro* cell experiments. The authors also did the *in vivo* test with magnetic resonance imaging (MRI) and photoacoustic imaging (PAI), showing the intriguing efficiency in anticancer and proving the safety of the AMF plus NIR-triggered dual-enhanced hyperthermia.

In another work, Cao et al. focused on cancer therapy with nanoengineering. They fabricated a biomimetic tumor-targeted black phosphorus (BP) nanoplatform to inhibit tumor cell growth. They found that the addition of oxaliplatin (1,2-diaminocyclohexane) platinum (II) (DACHPt) complexions would promote the efficiency of antitumor by photothermal effect and chemotoxicity. The mesenchymal stem cell (MSC)–derived membranes-based approach was introduced to circumvent the limitations of BP, including poor stability, dispersibility, low delivery sufficiency to the tumor site. By studying the cytotoxicity and antitumor efficacy of different nanoformulations under several conditions, this nanoplatform showed excellent tumor-targeted photothermal-chemo efficacy in cell tests. Nevertheless, *in vivo* study is encouraged to explore their antitumor potential further.

Xiong et al. reported another original research for cancer therapy with nanoengineering. They constructed novel star-shaped block polymers nanoparticles (NPs) containing Dacarbazine (DTIC), showing intriguing results against malignant melanoma (MM), which is one of the most life-threatening cancer. The introduction of these NPs brings the increased drug loading capacity of DTIC and also enables this DTIC with control release property. For the active targeted therapy of MM, the authors made further modifications with the nucleic acid aptamer AS1411. For validating the stability of this drug delivery system, the drug release profiles of DTIC, and the cell viability of NPs, they did several experiments *in vitro*. The results showed the great potential of this novel system. The antitumor efficacy and side effects of the constructed nanocarriers were evaluated by *In vivo* experiments, which demonstrated an intriguing effect to treat MM with virtually no side effects.

Nanoengineering also demonstrated potential in the area of bone-related diseases. In one original research article, a bilayer coating with double-crosslinked hydrogels containing bone morphogenic protein (BMP)-2 (alginate-GelMA/hydroxyapatite (HA)/BMP-2) was designed by Ma et al., showing intriguing potential in inducing osteogenesis of bone mesenchymal stem cells with the combination of alginate, GelMA, and BMP-2. The authors tested the biocompatibility, release of BMP-2, expression of the gene, and osteogenesis ability of this system with human bone mesenchymal stem cells (hBMSCs), finding the best ratios between GelMA and alginate for the applications in biology, like promoting the osteogenesis of the bone defect.

In this Research Topic, another application based on nanoengineering in bone-related diseases was explored for treating and preventing post-operative infections and bone loss. Yao et al. fabricated a nanosystem based on enoxacin (Eno)-loaded mesoporous silica nanoparticles (MSN). The eight repeating sequences of aspartate (D-Asp8) and polyethylene glycol were added to this nanosystem. The purpose of designing this system was to promote the translational practice of enoxacin in limiting post-operative infection and preventing bone loss by addressing the poor specificity, limited penetration into bone tissue, and adverse effects of enoxacin. Through *in vitro* and *in vivo* experiments, the biocompatibility, drug release performance, antibacterial properties, bone-targeting properties of this system were tested, which proved the potential in post-operative infections and bone loss.

In conclusion, the current Research Topic reports the novel design and nanoengineering of all kinds of drug delivery systems with various methods based on chemistry, biology, and materials science, providing new chances for developing advanced drug delivery systems towards clinical translation. The articles in this Research Topic will be a helpful reference, promoting the development of innovative nanoengineering approaches and accelerating the translation of related technology to clinical uses.
